# Precipitation Reaction of SDS and Potassium Salts in Flocculation of a Micronized Megestrol Acetate Suspension 

**Published:** 2013

**Authors:** Seyed Mahdi Hejazi, Mohammad Erfan, Seyed Alireza Mortazavi

**Affiliations:** *Department of Pharmaceutics, School of Pharmacy, Shahid Beheshti University of Medical Sciences, Tehran, Iran*.

**Keywords:** KCL-SDS Precipitation Reaction, Flocculation, HPMC-SDS Interaction, Megestrol Acetate, Suspensions

## Abstract

In this work attempts were made to evaluate K+-SDS and hydrocolloid polymer-SDS interactions in flocculation of megestrol acetate dispersions to enhance their stability as a part of suspension formulation. Different dispersions of micronized megestrol acetate and SDS were prepared. KCl and KH_2_PO_4_ and their corresponding sodium salts were added to the dispersions and the preparations were evaluated using general physicochemical and stability tests including appearance, sedimentation volume, sedimentation rate and redispersibility. Addition of polyols and hydrocolloid polymers to the SDS containing dispersions was also investigated for possible instabilities. SDS deflocculated the initial megestrol acetate dispersions. The use of potassium salts unlike the sodium salts flocculated the dispersion particles due to precipitation reaction of potassium ions and the adsorbed SDS. Additionally the uncharged hydrocolloid polymers MC and HPMC in contrast to the ionic polymers xanthan gum and NaCMC showed incompatibility due to their interaction with SDS. K^+^- SDS interactions have proved useful in protein and DNA analysis studies and we found this precipitation reaction to be applicable in flocculation of pharmaceutical suspensions containing SDS.

## Introduction

Phase separation in drug dispersions and the eventual caking of sediment is the major stability issue encountered in pharmaceutical suspension formulation. Preparation of physically stable suspensions includes the use of structured vehicles and the flocculation of the dispersion suspenoids. It is best to apply both these methods to prepare physically stable suspensions with uniform and elegant appearance (1). 

In a deflocculated dispersion, the particle charge exceeds the critical zeta potential, and therefore the repulsive forces between suspenoids supersede the London-Van der Waals attractive forces. The particles with supracolloidal sizes settle due to gravity and the impaction of the sediment by the weight of upper layers will over time lead to formation of compact and non-redispersible sediment called “Cake”. Flocculation has been defined as formation of voluminous and porous network structure of weakly bound particles that is resistant to caking and makes the dispersions easily redispersible (1, 2). 

Different agents have been studied for particle flocculation. This includes electrolytes, surfactants, hydrocolloid polymers and polyelectrolytes. Various mechanisms of flocculation in pharmaceutical suspensions have been proposed in the literature (1, 3-4). 

Flocculation by zeta potential reduction or charge neutralization is induced by decrease in the electrostatic repulsive barrier originating from the Double layer. The attractive forces of Van der Waals overwhelm and this results in particle aggregation. DLVO theory has been used to interpret this phenomenon in pharmaceutical suspensions (1, 3).

Chemical bridging has been described as aggregation by chemical interaction between the adsorbed ions extending from the particle surface and media precipitation ions and usually happens when a surfactant or macrogol has affinity to an electrolyte (2, 4). 

Flocculation can also be brought about by polymer or polyelectrolyte chains in low concentrations through formation of physical bridges. This is called bridging flocculation and it generally happens when high molecular weight polymer adsorbs on the surface without saturating it (5).

In liquid bridging, the flocculation is usually achieved by the use of low HLB surfactants and it can be described as follows: nonionic low HLB surfactants form hazy solutions which contain many large micelles. Upon incorporation of the suspension particles, a saturated layer of surfactants will form around the particles. This surrounding liquid links particles like a bridge, resulting in particle aggregation. It is thought that flocculation of suspensions by nonionic surfactants and polyols is related to dehydration of the particles and subsequent liquid bridging (6, 7).

SDS (sodium dodecyl sulfate) is an anionic surfactant with many applications in science and industry and it may be used as detergent, wetting agent, emulsifier, dispersant, or foaming agent. SDS has good wetting properties and it can act as a dispersant or a flocculating agent in suspensions and emulsions depending on the dispersion system characteristics (8). Its use in the DNA extraction is to aid in lysing cells and solubilizing proteins. In SDS-PAGE electrophoresis technique, it is used to unravel proteins to their primary conformation and impart a uniform charge density to the polypeptides. Aqueous solutions of SDS have also been useful to disperse and suspend nanotubes (9-11, 25). 

Megestrol acetate suspensions containing SDS have been seen to deflocculate in concentrations above 0.02% (w/v) making them unstable (12). SDS exhibits different interactions within the dispersion systems with polymers or other dispersion constituents. This may prove useful in certain circumstances. SDS reduces the deleterious effect of magnesium stearate on dissolution rate of the oral formulations which results from physical interaction of SDS and magnesium stearate during the mixing process (13). In SDS-hemoglobin analysis, the hydrophobic part of the SDS acts upon the globin subunit and the interaction of the hydrophilic part with oxidized iron subunit produces a stable reaction product that can be analyzed (14). Interaction of SDS micelles with Fe^3+^ and Al^3+^ has also been studied in adsorptive micellar flocculation (AMF) in which pollutant containing micelles can be flocculated and filtered out (15). In DNA extraction, SDS-KCl reaction has been employed to facilitate the separation of the SDS bound proteins (9). Unlike soaps, SDS is compatible with dilute acids and magnesium and calcium ions (8). In concentrations well above its *cmc *(critical micelle concentration), SDS induces particle aggregation in some dispersions through depletion flocculation (5). 

In view of numerous applications of the SDS and its dual efficacy both as a dispersing and an aggregating agent in the dispersion systems, attempts were made in the present work to study the interaction of the SDS-megestrol acetate dispersions with salts, polyols and polymers to examine the effect of SDS in flocculation of megestrol acetate suspensions.

## Experimental

Megestrol acetate was purchased from Pharmabios, Spain as micronized powder. Xanthan gum was obtained as a gift sample from Hakim Pharmaceutical Company, Iran. NaCMC, HPMC, MC, Glycerol, PEG 400, Sorbitol, Sodium Chloride, Potassium Chloride, Sodium Dihydrogen Phosphate, Potassium Dihydrogen Phosphate were all purchased from Merck, Germany. 

The suspensions were prepared as follows: polymer, electrolyte and the surfactant stock solution were made one day prior to suspension formulation. Polymer stock solutions were allowed to stand overnight to ensure adequate hydration. Surfactant solution was added to the calculated amount of megestrol acetate to make a concentrated uniform dispersion of megestrol acetate. The dispersion was later moved into a graduated cylinder and the electrolyte solution was added to produce flocculation. The dispersion was then promptly admixed into the polymer solution to obtain a uniform homogenous suspension. Each prepared suspension was moved to another container for stability observation.

*General tests performed*

Prepared suspensions were evaluated for their appearance, sedimentation volume, sedimentation rate and redispersibility. Sedimentation volume (SV or F) was the main parameter evaluated in this study and was measured as fraction of the total sediment volume. The results were expressed as percentage. Particle size analysis and zeta potential measurements were made using Malvern instruments.

*Wetting*

Using the sessile drop method, the contact angle of the distilled water on megestrol acetate was measured at well above 90 degrees. This is in agreement with the claimed high hydrophobicity of the megestrol acetate. SDS was used in this study as the wetting agent. Preparations of megestrol acetate dispersion with different concentrations of SDS were made in order to show how SDS affects the sedimentation volume.

*Flocculation*

NaCl, NaH_2_PO_4_ (NaDP), KCl and KH_2_PO_4_ (KDP) were used in different concentrations and the Sedimentation Volume (SV) was measured for the SDS-megestrol acetate dispersions after two weeks. Next, SDS was added incrementally in different preparations containing KCl and KH_2_PO_4_, and the sedimentation volume of the preparations made (18 formulations) was measured after two weeks. 

In another set of experiments, glycerol (G), propylene Glycol (PG), sorbitol and polyethylene glycol (PEG 400) were used to evaluate their effect on megestrol acetate dispersions containing SDS. 

*Polymers*

Hydrocolloid polymers including CMC, HPMC, MC and xanthan gum were incorporated in 0.1-0.6%(w/v) concentrations and the sedimentation volume of the preparations were compared and evaluated.

## Results

*Wetting*

**Figure 1:**
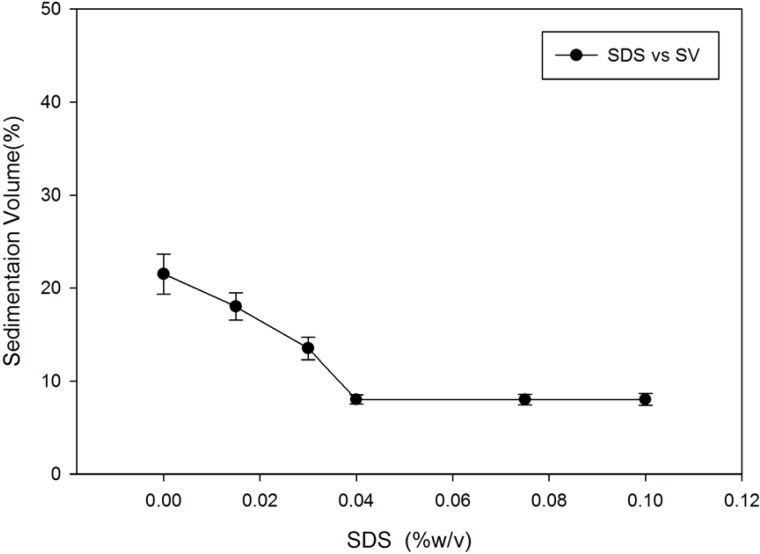
Effect of SDS on sedimentation volume of Megestrol acetate dispersions

Figure 1 shows the effect of SDS on sedimentation volume of megestrol acetate dispersion. It is evident that increasing the SDS concentration reduces the sedimentation volume. This effect can be attributed to negative charging of the particles that hinders particles from aggregation. The large particles will eventually settle and compact upon long term storage.

*Flocculation*

**Figure 2:**
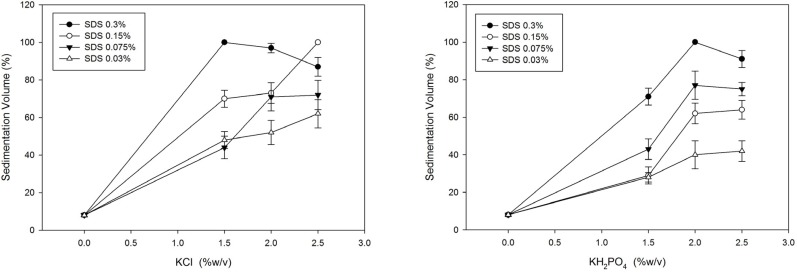
Effect of KCl and KH2PO4 on sedimentation volume of Megestrol acetate dispersions with varying amount of SDS

Sedimentation results after two weeks in dispersions containing electrolytes are presented in Table 1. It shows that potassium salts in contrast to sodium salts increase sedimentation volume. SDS was then used in different concentrations in dispersions containing KCl and KH_2_PO_4_. Formulations prepared in this step showed good sedimentation behavior and as can be seen in Figure 2, SDS increments noticeably increase the sedimentation volume and it seems to reach plateau at concentrations around 2-2.5%(w/v) of KCl or KH_2_PO_4_.

**Table 1:** Effect of different electrolytes on sedimentation volume of megestrol acetate dispersions with 0.02%(w/v) SDS. SV: Sedimentation Volume, KDP: KH_2_PO_4_, NaDP: NaH_2_PO_4_. (Values are means of 3 replicates ±SD, n=3).

**SV at 1.5% Electrolyte%Concentration **	**SV at 1% Electrolyte%Concentration **	**SV at 0.5% Electrolyte%Concentration**	**Electrolyte**
65±3.0	60±3.5	52±2.5	KCl
50±2.5	45±2.5	34±4.5	KDP
13±1.5	13±1.5	13±1.5	NaDP
13±2.0	13±2.0	13±2.0	NaCl

*Polyols and polymers*

At this step the polyols including glycerol, propylene glycol and sorbitol were used in different concentrations. Use of polyols resulted in high sedimentation volumes in the dispersion. PEG 400 produces deflocculated dispersions resulting in formation of compact sediment. 

Hydrophilic polymers including MC, HPMC, NaCMC and xanthan gum were used in two previously prepared formulations containing KCl and SDS to evaluate polymer effects on sedimentation volume and the results have been shown in Table 2. It can be seen that the suspensions with nonionic polymers (HPMC and MC) in contrast to the anionic ones (xanthan gum and NaCMC) are deflocculated with the sedimentation volume at around 8%.

**Table 2:** Effect of different polymers on sedimentation volume of megestrol acetate dispersions. SV: Sedimentation Volume. (Values are means of 3 replicates ±SD, n=3). A and B formulations represent the preparations containing different amount of SDS and electrolyte

**MC)SV%(**	**HPMC)SV%(**	**NaCMC)SV%(**	**Xanthan Gum)SV%(**	**Formulation**	**Polymer Concentration)w/v%( **
8±0.5	8±1.0	45±3.5	68±3.5	A	0.1
8±1.5	8±1.0	22±2.5	69±2.5	B	
8±0.5	8±0.5	55±1.5	86±3.0	A	0.2
8±1.0	8±1.0	33±4.5	92±1.5	B	
8±0.5	8±0.5	57±4.5	100±0.0	A	0.4
8±0.5	8±1.0	41±2.0	97±1.5	B	
8±1.0	8±0.5	58±4.5	100±0.0	A	0.6
8±0.5	8±1.0	47±3.0	100±0.0	B	

**Figure 3:**
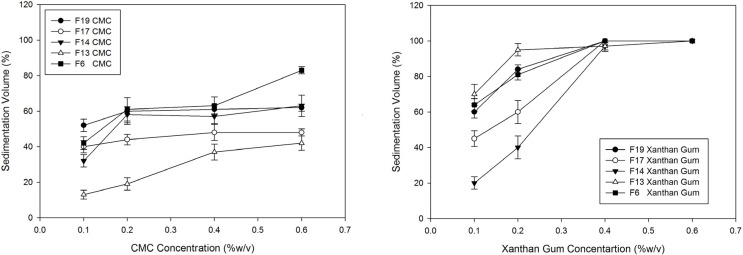
Effect of xanthan gum and CMC in different concentrations on sedimentation volume of Megestrol acetate dispersions

Figure 3 shows the better efficacy of xanthan gum in enhancing the dispersion sedimentation volumes compared with NaCMC. 

**Figure 4:**
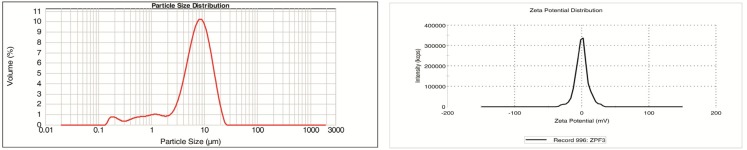
Particle size analysis (left) and zeta potential measurement (right) of a selected Megestrol acetate formulation containing SDS, KH2PO4 and Xanthan gum

Figure 4 shows the result of the particle size analysis and zeta potential measurement of a selected formulation with SDS 0.15%, KH_2_PO_4_ 2% and xanthan gum 0.2%.

## Discussion

Physically stable suspensions that do not form a caked deposit upon standing can be prepared by the use of structured vehicles and flocculation of the dispersion particles. To increase the sedimentation volume of the suspension, it is necessary to make a network structure in the system to prevent settling. This type of structurization can be produced by internal structurization (the structure produced by the dispersion medium or “microstructure”, *e.g. *flocculation) or external structurization (structure which is formed by the vehicle, *e.g*. viscosity imparting vehicles). 

Due to the hydrophobic nature of megestrol acetate, surfactants can be used to aid easy and uniform dispersion of the particles in the aqueous media. Surfactants may cause deflocculation of the dispersions. Deflocculation in the presence of ionic surfactants is typically due to the acquired high zeta potential of the particles (1). 

In the present work, effect of electrolyte, polyols and hydrocolloid polymers on SDS dispersions of megestrol acetate was studied. Considering the chemical structure of megestrol acetate particles in distilled water, H^+^ ions adsorb on the hydroxyl (–OH) functional groups and therefore the particles will be slightly positively charged. Addition of SDS and its subsequent surface adsorption imparts high negative charge to the particles. This will reduce the particle aggregation and phase separation occurs due the mass effect and settling of the suspenoids. 

SDS is incompatible with some alkaloidal salts and precipitates with lead and potassium salts. Calcium and magnesium salts do not show this property (8). SDS-KCl precipitation has proved useful in biological purification and protein analysis procedures. KCl or potassium acetate are employed to extract and purify SDS solubilized proteins and DNA structures (9, 16-17). We tested effect of such K+-SDS interaction in flocculating the SDS-megestrol acetate dispersions. Sodium salts were used as control and it was observed in the experiments that only potassium salts induce flocculation.

Upon addition of KCl, insoluble crystals of potassium dodecyl sulfate (KDS) form. When this happens, the surface charge of the megestrol acetate particles change and they no longer repulse each other due to the high negative charge. In fact the precipitation reaction brings about an alteration in surfactant films about the particles which unlike the surfactant film formed in previous steps, facilitates the linking of the particles allowing for aggregation of the suspenoids. 

Addition of polyols and hydrocolloid polymers to the megestrol acetate-SDS dispersions produced different results in terms of stability. The low molecular weight polyols glycerin, propylene glycol and sorbitol demonstrated no incompatibility and their use enhanced suspension appearance and stability. SDS was used at 0.02% in these preparations which is lower than the amount of SDS used in the dispersions containing potassium salts. The sedimentation volume observed was generally around 40-60% and was not as high as the sedimentation volume seen in the previous potassium salt containing suspensions with higher amounts of SDS.

Incorporation of HPMC, MC, and PEG resulted in defloccualtion in the dispersions containing SDS and potassium salts. The dispersions were therefore considered as unstable. On the other hand, K^+^-SDS dispersions of megestrol acetate with charged polymers of NaCMC and xanthan gum showed no compatibility issue and even the stability of such suspensions increased markedly due to the introduction of such polymers as viscosity enhancers. 

Addition of HPMC, MC and PEG possibly induces an interaction with the SDS in the dispersions. HPMC-SDS interactions have been studied in some recent works (18-20). In summary, in these studies an interaction region with specific concentration range for SDS and HPMC was identified in which such an interaction results in increased viscosity of the system. SDS forms complexes with the polymer chains of HPMC at the *cac *(critical association concentration) which is lower than *cmc *of the pure surfactant solution (21). The HPMC-SDS interaction in a preparation of a sunflower oil in water emulsion resulted in better emulsion stability and higher encapsulation efficiency after spray drying to produce the corresponding microcapsules (18). The SDS amounts used in our work were generally lower than the interaction region for HPMC and SDS found by the aforementioned studies; however, the high concentration of salts used in our experiments should also be factored in because the surfactant-polymer binding can be affected by salts. In one report for example, NaBr significantly decreased both the *cmc *and the *cac *of the SDS in presence of PVP and a related neutral copolymer (22). It is thought that in both the megestrol acetate suspension and the sunflower oil emulsion, the HPMC/SDS complex forms a more compact layer at the interface. This compact layer is usually considered favorable in emulsions as it hinders aggregation. However in the megestrol acetate dispersions in which the particle aggregation is desirable, such a HPMC/SDS complex formation results in defloccualtion and the subsequent sedimentation settling in the suspensions. 

SDS interactions with MC and PEG have also been studied in the literature and similar mechanism appears to be responsible for the observed deflocculation of the dispersions in our study (23, 24). Xanthan gum and NaCMC as expected showed no incompatibility with SDS due to their ionic charge which reduces the chance of such unwanted interactions with anionic surfactants. 

## Conclusion

As discussed previously, potassium salts can be used to induce flocculation of dispersions pre-wetted by SDS. SDS is soluble in water while KDS is less soluble in the aqueous media. Addition or presence of sodium ions does not induce flocculation while potassium ions cause the megestrol acetate particles to aggregate. We found that such interaction is useful to flocculate megestrol acetate-SDS dispersions. This approach could be universal and its utilization may prove effective in any similar dispersion systems. Another finding of this study is that the uncharged hydrocolloid polymers HPMC and MC reverse the flocculation produced in this manner. This is believed to be caused by formation of SDS/HPMC complex in the form of a compact layer at the interface which hinders particle aggregation.
